# Efficient protection of microorganisms for delivery to the intestinal tract by cellulose sulphate encapsulation

**DOI:** 10.1186/s12934-020-01465-3

**Published:** 2020-11-26

**Authors:** Walter H. Gunzburg, Myo Myint Aung, Pauline Toa, Shirelle Ng, Eliot Read, Wee Jin Tan, Eva Maria Brandtner, John Dangerfield, Brian Salmons

**Affiliations:** 1 Austrianova Singapore, 41 Science Park Road, #03-15 The Gemini, Singapore, 117610 Singapore; 2 Institute of Virology, Department of Pathobiology, University of Veterinary Medicine, 1210 Vienna, Austria; 3 VIVIT - Vorarlberg Institute for Vascular Investigation and Treatment, Feldkirch, Austria

**Keywords:** Probiotics, Gut microbiome dysbiosis, Microbiota, Encapsulation, Acid protection, Cellulose sulphate, Living cell encapsulation

## Abstract

**Background:**

Gut microbiota in humans and animals play an important role in health, aiding in digestion, regulation of the immune system and protection against pathogens. Changes or imbalances in the gut microbiota (dysbiosis) have been linked to a variety of local and systemic diseases, and there is growing evidence that restoring the balance of the microbiota by delivery of probiotic microorganisms can improve health. However, orally delivered probiotic microorganisms must survive transit through lethal highly acid conditions of the stomach and bile salts in the small intestine. Current methods to protect probiotic microorganisms are still not effective enough.

**Results:**

We have developed a cell encapsulation technology based on the natural polymer, cellulose sulphate (CS), that protects members of the microbiota from stomach acid and bile. Here we show that six commonly used probiotic strains (5 bacteria and 1 yeast) can be encapsulated within CS microspheres. These encapsulated strains survive low pH in vitro for at least 4 h without appreciable loss in viability as compared to their respective non-encapsulated counterparts. They also survive subsequent exposure to bile. The CS microspheres can be digested by cellulase at concentrations found in the human intestine, indicating one mechanism of release. Studies in mice that were fed CS encapsulated autofluorescing, commensal *E. coli* demonstrated release and colonization of the intestinal tract.

**Conclusion:**

Taken together, the data suggests that CS microencapsulation can protect bacteria and yeasts from viability losses due to stomach acid, allowing the use of lower oral doses of probiotics and microbiota, whilst ensuring good intestinal delivery and release.

## Background

The human gut microbiome, comprising the total genome of gut microbiota [1], plays a major role in facilitating host metabolism and is a major contributor to the regulation and maintenance of host physiology, immunity and the nervous system. Tiny alterations in the status and composition of the human microbiome can have tremendous effects, resulting in dysfunction of metabolic, immunological and nervous pathways, and contributing to a broad spectrum of diseases [1, 2]. A recent example specifically links a reduction in *Dialister* and *Coprococcus* species that synthesize the dopamine metabolite 3,4-dihydroxyphenylacetic acid with depression [3]. If the microbiome could be brought back into balance then such diseases could potentially be treated.

The oral delivery of probiotic microorganisms is one means of modulating the microbiota but relatively high doses are currently required [1]. Another, more challenging way to achieve rebalancing of the microbiome is fecal microbiota transplantation (FMT) and there are a number of ongoing clinical trials in this area [4].

Oral delivery of microbiota and probiotics has been hampered by the highly acidic stomach conditions, followed by exposure to bile [5] encountered during digestion coupled with the necessity for availability in the intestine [6]. Some bacteria show a high degree of acid resistance such as certain strains of *L. reuteri* [7], however most members of the microbiota are sensitive to pH 2 and it has been shown that pH is the major driver of microbial diversity in FMT [8].

Although acid protective coatings have been developed for drugs, these are generally not compatible with the growth and survival of living organisms like probiotics and other microbiota. Further, studies that have shown that high numbers of at least one hundred million (10^8^) viable probiotic bacteria must repeatedly reach the intestine for health benefits to be achieved for the patient [9] suggest that bacteria-compatible acid protective coatings must be effective in order to be able to deliver therapeutically relevant doses of microbiota or probiotics.

Moreover, the requirement for continued maintained therapeutic levels of microbiota requires regular bacterial consumption, as has been demonstrated in dose–response studies. In such studies, probiotics like *Lactobacillus rhamnosus GG* only transiently colonize the gastro-intestinal tract. It has been shown that fifteen days after terminating the administration of *L. rhamnosus GG* in adults, the probiotic bacterium could only be recovered from stool samples of 27% of the volunteers [10].

A major challenge to experimentally determining the best protection method for orally delivered microbiota is the correct choice of artificial gastric juice. The makeup of gastric juice varies between individuals and according to the type and amount of food ingested [11] and the presence of milk components has been shown to enhance the survival of bifidobacteria in simulated gastric juice [12]. Studies using artificial gastric juice containing lipids (L + AGJ) such as non-fat milk, glucose, yeast-extract, and cysteine (NGYC) medium show a reduction in free *L. acidophilus* of between 3.5 and 5.5 logs [13] at pH 2 over three hours, whereas use of a non-lipid containing artificial gastric juice (AGJ) results in a reproducible reduction of 6 [14] to 6.5 logs [5]. Other bacteria are even more sensitive and reduction in viability of 8.5 logs for *L. casei* and of more than 11 logs for *B. bifidum* have been cited after 2 h exposure to pH 2 in AGJ [15]. Perhaps even more important is proteolysis of bacteria by pepsin in the stomach [16]. Thus, the makeup of the artificial gastric juice used for testing survival of encapsulated bacteria has a huge effect.

**Fig. 1:**
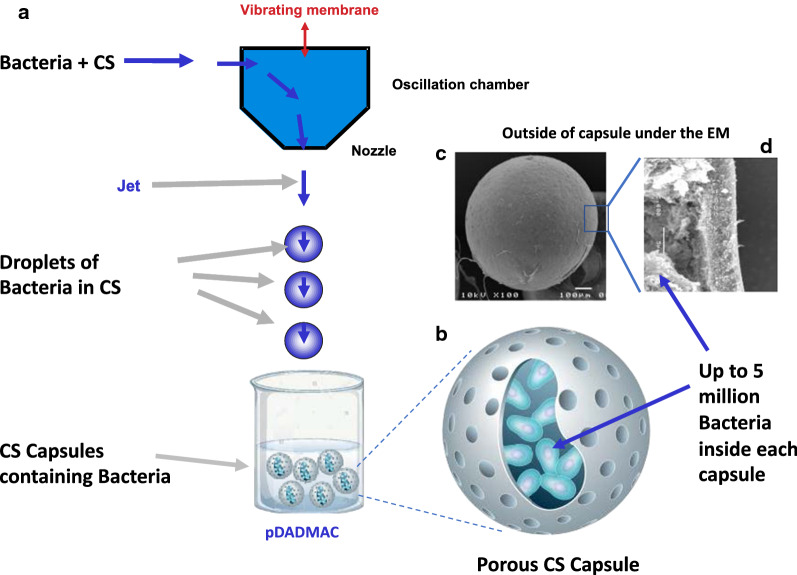
Cellulose sulphate encapsulation process. **a** Bacteria or yeast are mixed with Cellulose Sulphate (CS) and injected through a vibrating nozzle to form a stable jet of droplets which drop into the polymer pDADMAC. The droplets contain bacteria or yeast in the CS and harden as soon as they contact the pDADMAC to form capsules. After an appropriate hardening period, the capsules are washed. **b** The resulting capsules are porous and contain up to 5 million bacteria per capsule. **c** The capsules are regular and spherical in shape as evidenced by their appearance under the Scanning Electron Microscope, and when freeze fractured **d** reveal an outer related crust (consisting of CS and pDADMAC) and an inner space harboring the bacteria or yeast

We have developed a novel encapsulation method based on a simple extrusion technique using a modified form of cellulose in combination with poly-diallyldimethylammonium chloride (pDADMAC). The cellulose is plant derived and has been chemically modified by sulfation, conferring a negative charge [17]. Cellulose sulphate (CS) has been used previously to encapsulate mammalian cells but it has not been used for bacterial encapsulation [18]. Even though other cellulose derivatives have been used for coating in combination with other materials such as calcium alginate [19], or pectin derivatives [20] and in its carboxymethyl cellulose form with chitosan (CMC-Cht) hybrid micro- and macroparticles [21] or as bacterially produced cellulose as a carrier support [22] in the protection of probiotics, this is the first time CS has been used alone as an encapsulation material forming capsules in which the bacteria can grow and are protected. This is underscored by the fact that a recent review of the use of hydrogels for entrapment and protection of probiotics [23] makes no mention of CS. In our method (Fig. 1a), bacteria and yeast are encapsulated in CS at low density, become localized within the core of the CS capsule (Fig. 1b) and then are expanded post encapsulation by incubation of the capsules in appropriate medium to further increase the number of bacteria till the capsule is full, before being freeze dried and stored for long periods without cooling. The resulting encapsulated probiotics (Fig. 1c) are protected from low pH as found in the stomach and are released in the intestine where they are more efficient at colonization, presumably due to higher numbers of viable microorganisms reaching this site.

## Methods

### Bacteria growth and encapsulation

Most bacteria were obtained from the DSMZ (the German Collection of Microorganisms and Cell Cultures). *Lactobacillus acidophilus* (DMS 20079), *Lactobacillus johnsonii* (DMS 10533), *Lactobacillus casei* (DSM 20011) and *Bifidobacterium longum *subsp.* Infantis* (*B. infantis*) (DMS 20088) were grown in De Man, Rogosa and Sharpe (MRS) medium (Sigma). A genetically modified strain of *Escherichia coli K12 MG1655* (*E. coli-LUX*) was kindly provided by Mark Tangney and colleagues [24] and cultured in Luria (L) broth (Sigma). Our in house strain of *Saccharomyces boulardii* (officially classified as *Saccharomyces cerevisiae *var.* boulardii*) was grown in Yeast Extract–Peptone–Dextrose (YPD) medium. Overnight cultures of the bacteria or yeast (OD_600nm_ of 1) were pelleted by centrifugation at 4000×*g* for 4 min, washed in phosphate buffered saline (PBS), pelleted again and resuspended in 10 mL or 20 mL of 1.8% CS at a concentration of 2 × 10^6^ CFU/mL. The solution was put into a syringe and attached to a custom-built cell encapsulation machine which creates droplets of equal size (∅ = 0.7 mm). The droplets fall into a second solution, poly-diallyldimethylammonium chloride (pDADMAC), which is in excess and causes gelation of the droplets (Fig. 1a). After 2 min, the gelation was stopped by washing the capsules five times in excess volume of PBS. Typically, 30,000 capsules are produced per run at lab scale using this protocol. The generated capsules are characterized by size, number of bacteria or yeast, visual appearance and robustness. After encapsulation the capsules containing bacteria or yeast are cultured further using the same culture conditions as used for the starter culture prior to encapsulation for one or two days until the capsules are full (dependent on bacteria/yeast).

### Viability in acid followed by bile

Bacteria or yeast were cultured in appropriate media and then encapsulated as described above. Artificial gastric juice (AGJ) was produced by mixing HCl (pH 2), pepsin (10 g/L), NaCl (2.79 g/L), KCl (8.74 g/L), CaCl_2_ (0.24 g/L), glucose (77 g/L), glucosamine (33 g/L), lysozyme (1.52 g/L). Control gastric juice (CGJ) had the same composition as AGJ, except that the HCl, pepsin and lysozyme were not added. All components were supplied by Merck Millipore/Sigma Aldrich. Capsules were incubated for different times (1–4 h) in AGJ or CGJ. In certain experiments the capsules were then exposed to artificial bile according to Both et al. [25] for 1 h. Subsequently, encapsulated bacteria or yeast underwent decapsulation.

### Decapsulation

Bacteria or yeasts were de-encapsulated (decapsulation) using a decapsulation solution (Merck/Sigma-Aldrich-CIB002) that allows a cell-friendly dissociation of the capsule membrane and releases the cells alive into any medium of choice for further culture or processes such as cell counting. For decapsulation, 50 capsules were incubated with gentle agitation in 8 mL of decapsulation solution for 30 min at 37 °C × 50 rpm.

### Plate counting

The released bacteria were diluted in tenfold dilution steps in MRS medium, or L-broth (according to the bacteria as detailed above), or YTD medium (for *S. boulardii*), before being plated out on MRS or LB agar plates. The colonies arising were counted and then used to calculate the CFU/mL.

### Metabolic activity

Metabolic activity of the bacteria or yeast was determined using alamarBlue® assays designed to measure quantitatively the proliferation of various human and animal cell lines, bacteria and fungi according to the manufacturer’s instructions (Thermo Fisher Scientific DAL1025). Briefly, the assay measures the natural reducing power of living cells to convert resazurin, a cell permeable compound that is blue in colour and virtually non-fluorescent, into the bright red–fluorescent resorufin. The amount of fluorescence produced is proportional to the number of living cells. 10 µL of alamarBlue® was added into 100 µL of cell suspension and incubated for 2 h. The fluorescence of the alamarBlue® assay plate was read with a Tecan Infinite M200 reader using an excitation between 530–560 nm and an emission at 590 nm. Normalized metabolic activity was calculated as a percentage of the alamarBlue Relative Light Units (RLU) measured in the Tecan reader for the control, non-treated sample set as 100% compared to the RLU measured for the treated sample(s).

### Freeze-drying

The CS capsules were washed 5 times with 50 mL of fresh medium and resuspended in 20 mL appropriate incubation medium. 20 mL of freezing medium containing 5% milk powder, 1% glycerol, 10% trehalose was added, followed by incubation for 25 min at room temperature. Every 25 min, 20 mL of the incubating medium was replaced with 20 mL of fresh freezing medium and this was repeated 5 times. The medium was then removed and 1 mL of freezing medium added and the capsules plus medium transferred into 2R glass vials. The vials were then capped and shock-frozen in 100% ethanol and dry ice. The capsules were freeze dried using a commercially available freeze drying machine (Labconco Freeze Dryer, LBC#7400060). When the collecting chamber temperature of the freeze dryer reached − 80 °C, the vacuum pump was started and frozen vials with half-opened caps were placed into the freeze drying machine. Once freeze drying was completed, the caps were quickly closed and sealed with parafilm to ensure the vacuum and airtightness of the vials. The freeze-dried vials were stored at room temperature.

### Cellulase digestion assay

A range of different cellulase enzymes concentrations (10, 5, 1, 0.5, 0.1, 0.05, 0.01 EGU/mL) were tested using cellulase from *Trichoderma reesei* (Merck/Sigma-Aldrich-C2730) since it contains three enzyme components and is involved in the overall conversion process of cellulose to glucose. An EGU (Endoglucanase Unit) is measured relative to a Novozyme cellulase standard. The assay utilizes carboxymethyl cellulose (CMC) as the substrate and the assay is performed at 40 °C at pH 6.0.

Ten empty CS capsules were placed in each well of a 24-well plate. 2 mL of cellulase solution was added to 10 capsules for each sample, with each well receiving a different dose of cellulose (10, 5, 1, 0.5, 0.1, 0.05, 0.01 and 0.0 EGU/mL) in 50 mM sodium acetate. The plates were incubated at 37 °C with gentle agitation and examined every 30 min for the first 3 h and then after 8 h incubation as well as after overnight incubation.

### Testing of encapsulated bacteria in mice

A genetically modified strain of *E. coli K12 MG1655* kindly provided by Mark Tangney and colleagues [24] was used that had previously been shown to colonize the mouse gastrointestinal (GI) tract to high levels [26]. It carries the luxCDABE operon and constitutively auto-bioluminescence in the absence of exogenous substrate [27].

The *E. coli K12 MG1655* carrying the luxABCDE operon (*E. coli-LUX*) were cultured in Luria (L) broth. A 6 mL aliquot of the culture (OD_600nm_ of 1) was pelleted and resuspended in 1.8% cellulose sulphate for encapsulation. The CS capsules were incubated in L broth overnight. They were then freeze dried in 2R vials (1000 capsules/vial).

Two groups of male nude mice (Charles River/ Nu-FOXn1^nu^) that had been acclimatized for a week and fed LabDiet® 5001 Rodent Diet (Purina Mills, Inc., St. Louis, MO) ad libitum, received two different concentrations [2.7 × 10^9^ CFU (dose 1) or 5.4 × 10^9^ CFU (dose 2)] of non-encapsulated *E. coli-LUX* or encapsulated *E. coli-LUX* [28, 29] administered in 600 µL of saline which was orally dosed by gavage. Fecal pellets were collected 2 h, 4 h and 24 h post gavage. At 24 h after gavage of encapsulated or non-encapsulated *E. coli-LUX*, the animals were euthanized. After the necropsy, the stomach, cecum and colon were harvested. The organs and fecal pellets were subjected to bioluminescence imaging using an IVIS 200 spectrum (Perkin Elmer) imaging system. The luminescent exposure time was optimized and the samples were exposed to the emission spectrum of luciferase for 5, 1, and 0.5 s. The tissue samples and feces were exposed to the emission spectrum of luciferase for 10 s, 1, and 2 min. The bioluminescence was measured with an open filter. The signal was visualized as pseudo color images indicating light intensity (red being the most intense and blue the least intense), which are superimposed over the grayscale reference photographs. The images were analyzed by Living Image 4.4 software.

All of the animal experiments were conducted at Comparative Biosciences, Inc., California, USA, according to the regulations and guidelines for animal care and approved by the institutional animal care and use committee (IACUC#1298-1115).

## Results

To evaluate the generality of the use of this new cellulose sulphate based delivery method, five different strains of probiotic bacteria (*L. acidophilus*, *L. johnsonii*, *L. casei*, *L. casei shirota* and *B. infantis*) were encapsulated in CS (Fig. 1a) and all survived the encapsulation process with good viability (60–70% for *L. acidophilus* and *L. johnsonii*, 90–100% for *L. casei* and *B. infantis—*results not shown). Good viability was also observed for other strains of probiotic bacteria obtained from the DSMZ including *Lactobacillus plantarum *subsp.* plantarum* (DSM 20174)*, Lactobacillus paracasei *subsp.* paracasei* (DSM 2312)*, Bifidobacterium animalis *subsp.* lactis* (DSM 10140) and *Lactobacillus amylolyticus* (DSM 11664) (data not shown), indicating that the CS is not toxic for any strains of bacteria and yeast analyzed so far. Each CS capsule has a diameter of 0.7 mm and contains on average approximately 5 million *L. casei*, or 0.5 million *L. acidophilus* and *B. infantis* when full (after growth of bacteria within the capsule). The bacteria or yeast containing capsules (Fig. 1b) are porous. Scanning Electron Microscopy of the capsules reveals a round shape with some indentations (Fig. 1c). Freeze-fracture of the capsules (Fig. 1d) reveals an outer related layer with thickness of about 5 µm, surrounding a space in which the cells are located [30, 31]. The encapsulation process can also be adjusted so that capsules of a defined and reproducible size (with either increased or decreased diameter) can be produced (data not shown).

**Fig. 2:**
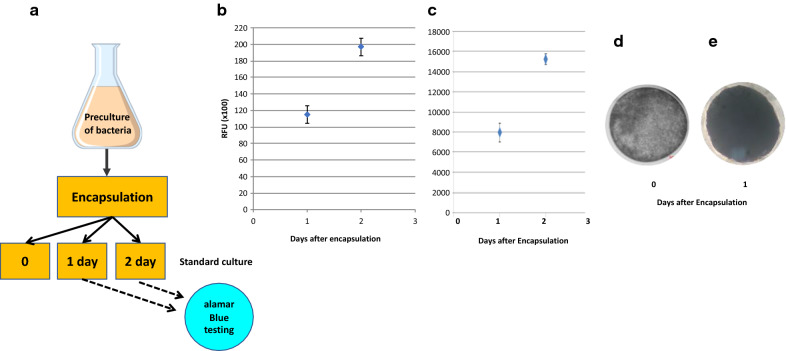
Growth and survival of bacteria post encapsulation. **a** After encapsulation of overnight pre-cultures of bacteria or yeast at fairly low bacterial density (2 × 10^6^ CFU/mL), the CS capsules containing the bacteria were incubated under standard bacterial growth conditions (appropriate medium and temperature with agitation) for 1 or 2 days to allow the encapsulated bacteria to multiply. Using the alamarBlue® assay, the metabolic activity of the bacteria was measured 1 day and 2 days after encapsulation and the Relative Light Units (RLU) recorded from the alamarBlue® assay plotted for *L. casei* (**b**) and for *E. coli K12* (**c**) Shown is the average of two experiments and the standard deviation for both bacteria. The ability of the bacteria to grow in the capsules was also visually evident when comparing the *L. casei* capsules immediately after encapsulation (**d**) with the *L. casei* containing capsules 24 h later (**e**) under the microscope (× 100 magnification)

After encapsulation at fairly low bacterial density (2 × 10^6^ CFU/mL), the CS capsules containing the bacteria (Fig. 2a) are incubated under standard bacterial growth conditions (appropriate medium and temperature with agitation) for 0, 1 or 2 days to allow the encapsulated bacteria to multiply. Experiments in which alamarBlue® metabolic activity assays were carried out at various time points after encapsulation (Fig. 2a) revealed that the bacteria increased in number within the capsule within hours. As an example, the metabolic activity (expressed as Relative Light Units, RLU) was determined in capsules containing *L. casei* one and two days after encapsulation (Fig. 2b). Over this 24 h period the metabolic activity in the capsules increased by 80% suggesting that the bacterial cell numbers had almost doubled. Similar results were obtained for all other bacteria or yeast encapsulated. As an example, Fig. 2c shows a similar increase in metabolic activity for *E. coli K12.* This was also visually evident when comparing the *L. casei* capsules immediately after encapsulation (Fig. 2d) with the capsules 24 h later (Fig. 2e).

**Fig. 3:**
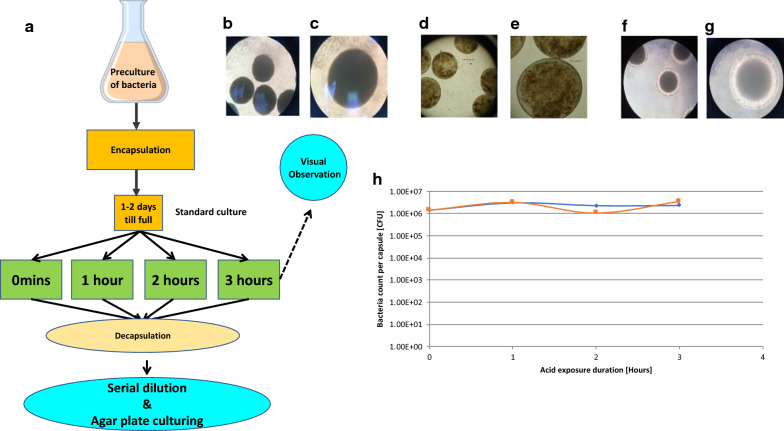
Resistance of encapsulated bacteria to Artificial Gastric Juice (AGJ). **a** After encapsulation of overnight pre-cultures of bacteria at fairly low bacterial density (2 × 10^6^ CFU/mL), the CS capsules containing *L. casei* (**b**, **c**), *L. acidophilus* (**d**, **e**) and *B. infantis* (**f**, **g**), were incubated under standard bacterial growth conditions (appropriate medium and temperature with agitation) for 1 or 2 days to allow the encapsulated bacteria to multiply. The encapsulated bacteria were then exposed to AGJ + P for 1, 2 or 3 h. The capsules were microscopically observed at ×40 (**b**, **d** and **f**) or ×100 (**c**, **e** and **g**) magnification after three hours exposure to AGJ + P. Encapsulated *L. casei* were decapsulated without exposure (0 min), or after 1, 2 and 3 h exposure to AGJ, submitted to limiting titration and plated on MRS agar plates **(h)**. The titres of decapsulated *L. casei* measured as CFU/capsule after exposure of the encapsulated *L. casei* to AGJ + P (blue diamonds, blue line) or to MRS (orange squares, orange line) are shown

To evaluate whether the CS capsules could provide an effective protection against the killing of the encapsulated bacteria by stomach acid, *L. casei* were encapsulated and cultured for 1 to 2 days post encapsulation till the capsules were full (Fig. 3a). The capsules containing *L. casei* were then exposed to Artificial Gastric Juice (AGJ) supplemented with pepsin and lysozyme (AGJ + P) for up to three hours or not exposed (0 min). After exposure of encapsulated *L. casei* for 3 h to AGJ + P at pH 2, microscopic analysis clearly showed that the capsules remained intact with no deformation (Fig. 3b) even at high magnifications (Fig. 3c).

Similar results were obtained for all other bacteria or yeast encapsulated. As an example *L. acidophilus* (Fig. 3d, e) and *B. infantis* (Fig. 3f, g) containing capsules are shown after analogous AGJ + P exposure at low (Fig. 3d, f) and high (Fig. 3e, g) magnifications. These results show that acid exposure even for 3 h did not affect the integrity of the capsules (compare with non-acid exposed *L. casei* containing capsules shown in Fig. 2d, e).

CS capsules containing *L. casei* were recovered immediately (0 min) or after 1, 2 or 3 h exposure to AGJ + P at pH 2 and the capsules dissolved using a decapsulation solution that releases the bacteria alive. After serial dilution in MRS medium and plating out on MRS agar plates (Fig. 3a), the growth of decapsulated bacteria exposed to AGJ + P at pH 2 for up to 3 h (Fig. 3h blue diamonds) is no different to the growth of decapsulated bacteria cultured in MRS throughout and not exposed to AGJ + P (Fig. 3h orange squares).

**Fig. 4:**
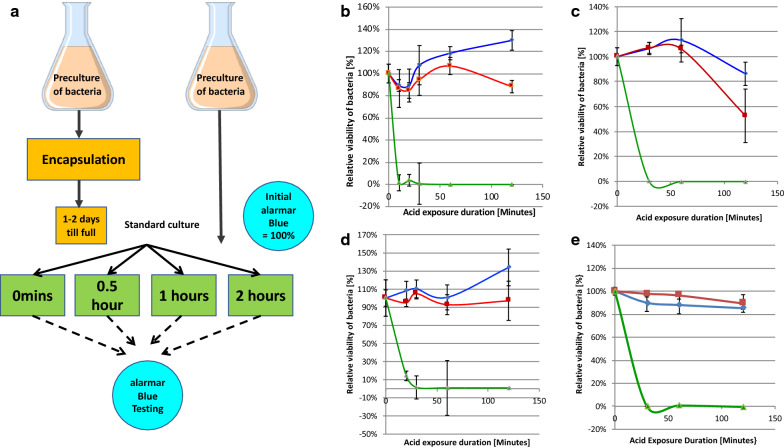
Relative viability of non-encapsulated versus encapsulated bacteria after exposure to Artificial Gastric Juice (AGJ). **a** After overnight culture of bacteria, or encapsulation of overnight pre-cultures of bacteria at fairly low bacterial density (2 × 10^6^ CFU/mL), the free or CS encapsulated *L. acidophilus* (**b**), *L. johnsonii* (**c**), *B. infantis* (**d**) and *L. casei shirota* (**e**) were incubated under standard bacterial growth conditions (appropriate medium and temperature with agitation) for 1 or 2 days to allow the bacteria to multiply. The viability of the bacteria was then measured in an AlamarBlue® assay. The relative viability of each bacterial species, free or encapsulated, was set at 100% and all subsequent measured viabilities calculated as a relative percentage to this initial 100%. The free (black up-pointing triangle—green lines, filled diamond—blue lines) and encapsulated (filled square—red lines) bacteria were then exposed to AGJ + P (black up-pointing triangle —green lines, filled square—red lines) or to AGJ without acid (filled diamond—blue lines) for 1, 2 or 3 h before being subjected to alamarBlue® metabolic activity measurement. **b–e** Time course of the relative viability of encapsulated (filled square—red lines) or free, non-encapsulated (black up-pointing triangle—green lines) *L. acidophilus* (**b**), *L. johnsonii* (**c**), *B. infantis* (**d**) and *L. casei shirota* (**e**) expressed as a percentage of the initial viability set as 100%, after 2 h exposure to artificial gastric juice plus pepsin and lysozyme (AGJ + P). For comparison the time course of viability of free bacteria (filled diamond—blue lines) *L. acidophilus* (**b**), *L. johnsonii* (**c**), *B. infantis* (**d**) and *L. casei shirota* (**e**) after 2 h exposure to artificial gastric juice without acid (AGJ) is also shown. The mean and the standard deviation are indicated

In a quantitative evaluation of metabolic activity as a surrogate for bacterial number, comparing four different bacteria to demonstrate the generality of the observations, bacteria were either encapsulated and then allowed to grow to fill the capsules over two days, or left non-encapsulated. The relative viability of encapsulated or non-encapsulated bacteria was determined using the indirect metabolic alamarBlue® Assay and initial metabolic activities normalized and set to 100% (Fig. 4a). The non-encapsulated and encapsulated bacteria were then exposed to AGJ + P or AGJ for different times before the relative viability was again determined using the alarmarBlue® Assay (Fig. 4a). Free, non-encapsulated (black up-pointing triangle—green lines) or encapsulated (filled square—red lines) *L. acidophilus* (Fig. 4b), *L. johnsonii* (Fig. 4c), *B. infantis* (Fig. 4d) and *L. casei shirota* (Fig. 4E) were exposed to AGJ + P at pH 2 for 3 min, 0.5 h, 1 h and 2 h and the viability after AGJ + P exposure plotted as a percentage of the initial viability (before exposure). The viability of the bacteria in AGJ without pepsin or acid was also measured (filled diamond—blue lines). The results showed that all four strains of encapsulated probiotic bacteria (red lines) survived AGJ + P at pH 2 better than non-encapsulated bacteria (green lines), where viability was reduced to undetectable levels after 30 min for all four bacteria (Fig. 4a–d).

**Fig. 5:**
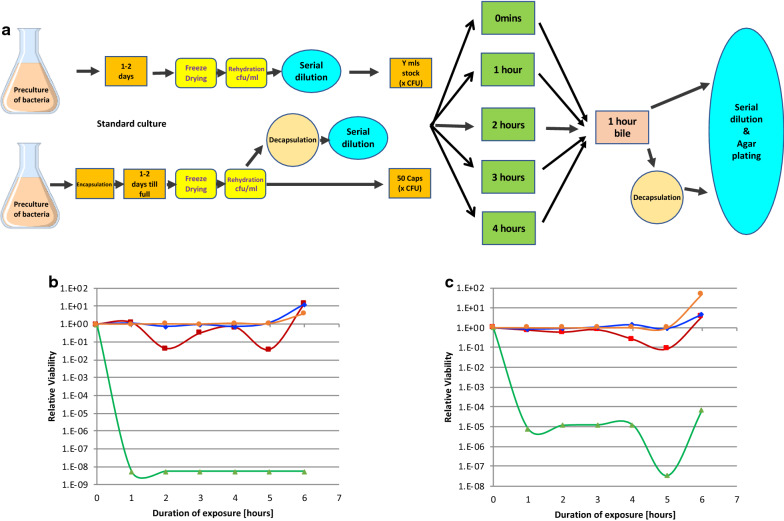
Survival of Encapsulated Bacteria and Yeast after acid exposure followed by bile. **a** After overnight culture of *L. casei* (**b**) or *S. boulardii* (**c**), or encapsulation of overnight pre-cultures, the free or CS encapsulated *L. casei* (**b**) or *S. boulardii* (**c**) were freeze dried and stored before rehydration and either direct titration or decapsulation followed by titration to determine the CFU per mL or per capsule. The CFU per 50 capsules was set as 1, and equivalent CFU of non-encapsulated bacteria or yeast also used in the “free, non-encapsulated” samples. 50 capsules or the equivalent CFU of non-encapsulated *L. casei* (**b**) or *S. boulardii* (**c**) was then subjected to exposure to AGJ + P (filled square—red lines, black up-pointing triangle—green lines), or to AGJ at pH 7 (filled diamond—blue lines, filled circle—orange lines) for up to 4 h, followed by exposure to artificial bile for 1 h. This was followed either by direct titration, or titration after decapsulation, on appropriate agar plates. The resulting measured CFU were expressed as relative viability compared to the initial CFU count (before acid or bile exposure) that was set as 1. **b** and **c** Time course of normalized survival of encapsulated (filled square—red lines) or free, non-encapsulated (black up-pointing triangle—green lines) *L. casei* (**b**), and *S. boulardii* (**c** after up to 4 h exposure to artificial gastric juice plus pepsin and lysozyme (AGJ + P) followed by one hour exposure to artificial bile. For comparison the time course of survival of encapsulated (black up-pointing triangle—blue lines) or free, non-encapsulated (filled circle—orange lines) *L. casei* (**b**), and *S. boulardii* (**c**) after 4 h exposure to artificial gastric juice at pH 7 (AGJ) followed by one hour exposure to artificial bile is also shown

In a second set of experiments, *L. casei* as an exemplar bacteria and *Saccharomyces boulardii* as an exemplar yeast were used. The resistance of non-encapsulated freeze dried bacteria or yeast, or bacteria or yeast encapsulated in CS, allowed to grow to fill the capsules, and then freeze dried to mimic the normal formulation of a commercial bacteria or yeast preparation as a freeze dried powder, was evaluated over a 4 h period in AGJ + P at pH 2, the mean fasting retention time in the stomach [32]. This was followed by one hour exposure to bile. Normalized CFU of freeze dried encapsulated (filled square—red lines) or non-encapsulated (black up-pointing triangle—green lines) *L. casei* (Fig. 5b) or *Saccharomyces boulardii* (Fig. 5c) were exposed to AGJ + P at pH 2 for four hours, followed by exposure for 1 h to bile and the number of surviving bacteria or yeast was determined after decapsulation, serial dilution and titration on agar plates. Results were plotted as the change in Relative Viability over time based on an initial Relative Viability set as 1. The viability of the free, non-encapsulated bacteria or yeast in AGJ without pepsin or acid was also measured (filled circle—orange lines), as was the viability of encapsulated bacteria or yeast exposed to AGJ at pH 7 (filled diamond—blue lines) and showed no overall changes in viability over the course of the experiment. The viability of non-encapsulated *L. casei* was reduced ~ 8 logs within 1 h exposure to AGJ + P (Fig. 5b. black up-pointing triangle—green line 1 h point) whereas encapsulated *L. casei* exposed to AGJ + P at pH 2 for 4 h, followed by 1 h bile exposure showed no significant effect (Fig. 5b. filled square—red line average of 5 and 6 h points). Similarly, the viability of non-encapsulated *S. boulardii* was reduced ~ 5 logs within 1 h exposure to AGJ + P (Fig. 5c. black up-pointing triangle—green line 1 h point) whereas encapsulated *S. boulardii* exposed to AGJ + P at pH 2 for 4 h, followed by 1 h bile exposure showed no significant effect (Fig. 5c. filled square—red line average of 5 and 6 h points). In both cases the addition of bile juice to the encapsulated microbiota caused a transient reduction in cell number followed by recovery within the next hour.

To evaluate whether encapsulated bacteria were released after transit through the stomach and intestine as a result of a combination of the presence of low amounts of active cellulase produced by representatives of *Bacillus* genus in the human gastrointestinal tract [33, 34], and peristaltic movement causing breakage or bursting of the capsules, both in vitro and in vivo experiments were carried out.

**Fig. 6:**
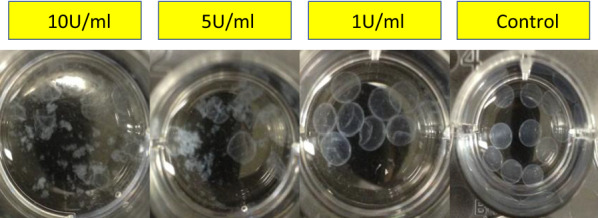
Release of Encapsulated Bacteria in vitro. Capsules were incubated in three (10 U/mL, 5 U/mL, 1 U/mL) concentrations of cellulase with gentle shaking overnight and visual disintegration of the capsules documented. The control was shaken gently overnight without the addition of cellulase

To demonstrate release under these conditions in vitro, CS capsules were incubated at room temperature with gentle shaking in various concentrations of cellulose chosen to reflect those produced by commensal *Bacillus* genus in the human gastrointestinal tract [33, 34]. Figure 6 shows visually the effects of overnight incubation and shaking without cellulase (Control), and with increasing amounts of cellulase (1 U/mL, 5 U/mL and 10 U/mL). Incubation with 10 U/mL cellulase and overnight shaking caused the capsules to visually disintegrate (Fig. 6). Table 1 shows the results of the complete experiment in which cellulase concentrations between 0.01 U/mL and 10 U/mL were tested with or without touch and after incubation for between 1 h and overnight. Cellulase concentrations of 0.05 U/mL were sufficient to cause capsule disruption (+) on touch after 8 h (Table 1), whilst even concentrations as low as 0.01 U/mL caused capsule disruption (+) on touch after overnight incubation.

**Table 1:** Effect of various cellulase concentrations and overnight incubation with shaking on capsule stability

Incubation time	Observation	Cellulase concentration					
		10 U/mL	1 U/mL	0.5 U/mL	0.1 U/mL	0.05 U/mL	0.01 U/mL
1 h	Debris	+	−	−	−	−	−
	Burst on touch	+	−	−	−	−	−
2 h	Debris	+	+	−	−	−	−
	Burst on touch	+	−	−	−	−	−
3 h	Debris	++	+	−	−	−	−
	Burst on touch	+	+	−	−	−	−
8 h	Debris	+++	+	−	−	−	−
	Burst on touch	+	+	+	+	+	−
Overnight	Debris	++++	+	−	−	−	−
	Burst on touch	+	+	+	+	+	+

Debris: − no debris; + detectable debris; ++ major debris; +++ most capsules as debris; +++ all capsules as debris

Burst on touch: − no; + yes

**Fig. 7:**
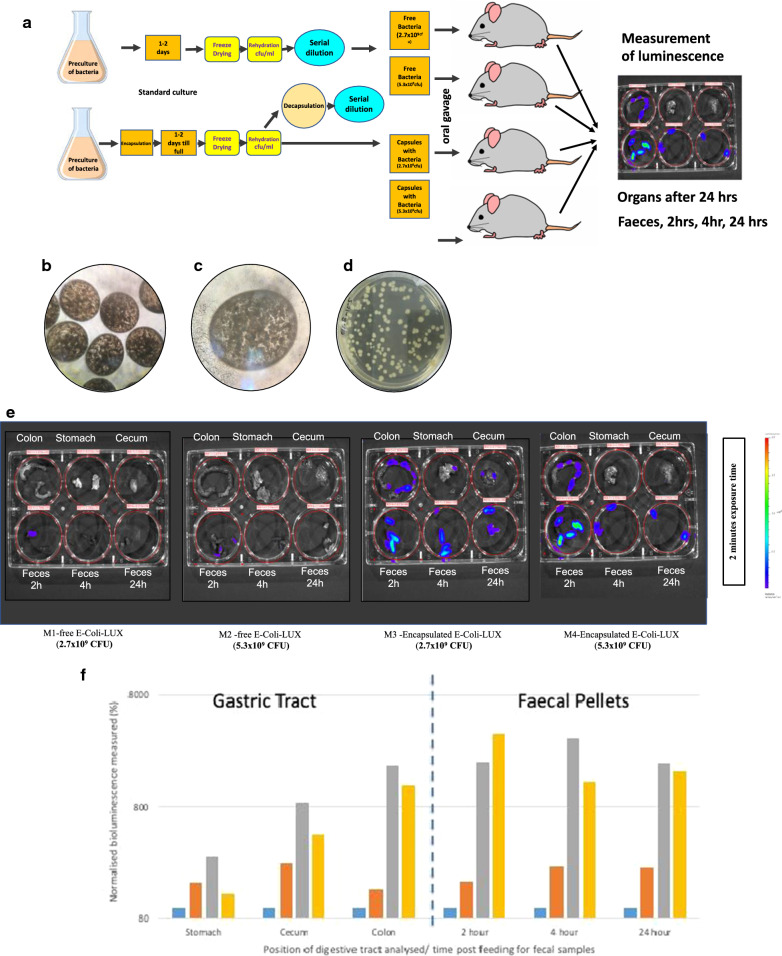
Release of encapsulated bacteria in vivo. **a** After overnight culture of *E. coli-LUX* or encapsulation of overnight pre-cultures, the free or CS encapsulated *E. coli-LUX* were freeze dried and stored before rehydration and either direct titration or decapsulation followed by titration to determine the CFU per mL or per capsule. *E. coli-LUX* containing capsules after rehydration are shown in (**b**) ×40 magnification and (**c**) ×100 magnification. After decapsulation the *E. coli-LUX* bacteria were plated on agar plates and the titre determined (**d**)*.* The number of capsules or amount of free bacteria equivalent to 2.7 × 10^9^ CFU or 5.3 × 10^9^ CFU was administered to mice by oral gavage. 2 h, 4 h and 24 h post gavage feces were harvested and 24 h after gavage the animals were euthanized and stomach, cecum and colon harvested. These organs, as well as the feces were placed in individual wells of six well plates and exposed to the emission spectrum of luciferase for 10 s, 1, and 2 min. **e** Four mice were administered 2.7 × 10^9^ CFU of free *E. coli-LUX* (M1) (left most six well plate), 5.3 × 10^9^ CFU of free *E. coli-LU*X (M2) (six well plate second from left), 2.7 × 10^9^ CFU of encapsulated *E. coli-LUX* (M3) (six well plate third from left) or 5.3 × 10^9^ CFU of encapsulated *E. coli-LUX* (M4) (rightmost six well plate) by oral gavage. 2 h, 4 h and 24 h post gavage feces were harvested and 24 h after gavage the animals were euthanized and stomach, cecum and colon harvested. These organs, as well as the feces were placed in individual wells of six well plates and exposed to the emission spectrum of luciferase for 10 s, 1, and 2 min. Here the results from 2 min exposure are shown. The bioluminescence was measured with an open filter. The signal was visualized as pseudo color images indicating light intensity (red being the most intense and blue the least intense), which are superimposed over the grayscale reference photographs. **f** The bioluminescence signal from the gavage experiment described above was quantitated using Living Image 4.4 software. The signal from stomach, cecum, colon, 2 h feces, 4 h feces and 24 h feces from mice administered 2.7 × 10^9^ CFU of free *E. coli-LUX* (M1) (blue bars), 5.3 × 10^9^ CFU of free *E. coli-LUX* (M2) (orange bars), 2.7 × 10^9^ CFU of encapsulated *E. coli-LUX* (M3) (grey bars) or 5.3 × 10^9^ CFU of encapsulated *E. coli-LUX* (M4) (yellow bars) is shown.

To confirm the in vitro observations that encapsulated bacteria are protected from acid and bile exposure and can be released by the action of cellulases in the lower intestine, two different concentration of non-encapsulated *E. coli-LUX* or encapsulated *E. coli-LUX* were administrated to mice by the gavage technique (Fig. 7a). Briefly, freeze dried capsules containing *E. coli-LUX* (Fig. 7b, c) were rehydrated and decapsulated before being subjected to serial dilution and plating out (Fig. 7d). The number of bacteria per capsule was determined, and the number of capsules calculated that contained either 2.7 × 10^9^ CFU or 5.3 × 10^9^ CFU. In parallel, free non-encapsulated *E. coli-LUX* that had also been freeze dried and rehydrated were titrated and the volume containing either 2.7 × 10^9^ CFU or 5.3 × 10^9^ CFU calculated. *E. coli-LUX* have previously been shown to colonize the mouse gastrointestinal (GI) tract to high levels [26], carry the luxCDABE operon and constitutively auto-luminesce in the absence of exogenous substrate [27]. *E. coli-LUX* were chosen to allow clear identification and differentiation of the encapsulated bacteria compared to commensal bacteria already present in the mouse which are needed to enable the testing of commensal bacteria cellulase- mediated release of the encapsulated bacteria. Either 2.7 × 10^9^ CFU or 5.3 × 10^9^ CFU *E. coli-LUX* were administered to nude mice, either as free bacteria, or in capsules, by oral gavage. There was no lethality and no untoward observations of toxicity during the duration of the study. After 24 h, mice were euthanized. No significant observations were recorded at necropsy. Organs and feces were collected and placed individually in wells of multi-well plates (Fig. 7a).

Figure 7e shows the intensity of the bioluminescent signal from colon (upper left well), stomach (upper center well), cecum (upper right well), feces 2 h post gavage (lower left well), feces 4 h post gavage (lower center well), feces 24 h post gavage (lower right well) in mice fed 2.7 × 10^9^ CFU free *E. coli-LUX* (left most plate, M1), 5.3 × 10^9^ CFU free *E. coli-LUX* (second from left plate, M2), 2.7 × 10^9^ CFU encapsulated *E. coli-LUX* (third from left plate, M3) and 5.3 × 10^9^ CFU encapsulated *E. coli-LUX* (right most plate, M4). The bioluminescent signal was not detectable in the tissue samples collected from mice treated with non-encapsulated *E. coli-LUX* (top rows of two left most plates), and only in the 2 h feces from non-encapsulated *E. coli-LUX* (left most well on bottom row of two left most plates). In contrast, a clear bioluminescent signal was seen in the colon of mice treated with encapsulated *E. coli-LUX* (top left wells of the two rightmost plates). Similarly, the collected feces after 2, 4 and 24 h showed detectable bioluminescent signal in the mice treated with encapsulated *E. coli-LUX* (bottom wells of the two rightmost plates).

The bioluminescent signal was quantitated after various timepoints of exposure and the quantitative analysis is shown in Fig. 7f. The signal was detectable mostly in the colon and feces of mice treated with encapsulated *E. coli-LUX*. Figure 7f shows similar amounts of bacteria were found to have remained in the stomach 24 h after gavage of marked bacteria regardless of whether they were encapsulated or not (Fig. 7f), however more bacteria were found in the cecum in those mice receiving encapsulated rather than non-encapsulated bacteria and this difference was even more marked and more than 1 log higher in the large intestine (colon). Similar differences in amounts of living bacteria were also seen in fecal pellets 2 and 4 h post-gavage as well as 24 h after gavage (Fig. 7f). GI transit in a mouse is around 4–6 h [35–37]. Thus, the data suggests that not only are the encapsulated bacteria protected from acid destruction during passage through the stomach, but additionally there is release and colonization of the intestine as evidenced by the continued presence of marked bacteria in the feces at a constant level even after 24 h.

## Discussion

Many attempts have been made to protect probiotics during passage through the GI, but none of these methods have been very effective. A recent review of protection offered to probiotics by various coatings [38] reveals that encapsulation with the de facto industry standard, alginate, followed by exposure at pH 1.8 in AGJ but with pepsin (AGJ + P) still results in loss of 10 logs activity after 90 min for *L. plantarum* [39], and of at least 9 logs for *L. brevis* after 2 h even in the absence of pepsin (AJG) [40].

A secondary coating of chitosan has been shown to increase the acid resistance of *B. breve* in alginate capsules by around 4.5 logs [40] in AGJ pH 2 for 2 h, however the overall viability is still reduced by at least 4 logs. Similar results have been reported for *L. casei* and *B. bifidum* where a coating of chitosan was applied to alginate-gelatinized starch capsules and resulted in an increase in acid resistance (compared to alginate-gelatinized starch alone) of almost 1 log. However, the overall viability after 2 h in AGJ + P is still reduced by 4 to 5 logs [15]. Use of AGJ also resulted in a reduction of overall viability by 2.5 to 3 logs for *L. acidophilus* and of 3.5 to 4 logs for *L. casei* after 2 h exposure of the alginate chitosan coated capsules at pH 1.55 [14].

A secondary whey protein coating has also been applied to alginate capsules and shown to increase the resistance of encapsulated *L. plantarum* to acid in AGJ + P by 5 to 7 logs, however overall viability is still reduced by 3 to 5 logs after 2 h [39].

Use of poly-l-lysine (PLL) to coat the alginate encapsulated *L. acidophilus* or *L. casei* has less of a protective effect after exposure to AGJ at pH 1.55 for two hours with losses in viability of 4–5 logs and of 5–6 logs respectively [14]. In another study, losses of viability of around 3 logs have been shown for alginate capsules coated with palm oil and PLL exposed to AGJ at pH 2 for two hours for a wide variety of bacteria (*L. rhamnosus*, *L. salivarius*, *L. plantarum*, *L. paracasei*, *B. infantis* and *B. lactis*), whilst *L. acidophilus* only showed a loss of 2 logs [41].

Most recently, a study has shown that a layer-by-layer approach using chitosan, followed by alginate and repeated (LbL − (CHI/ALG)2) and even a multi-layered Chitosan capsule alone (LbL − (CHI/L100)2) can afford effective protection against pH 2 over two hours with only loss of 1 log in viability in AGJ [42]. However, this study was conducted in the absence of pepsin.

Thus, there is still a need to find simple high efficiency methods to protect bacteria delivered by the oral route from gastric conditions including enzymatic destruction by pepsin and lysozyme.

We have shown here, for a number of commonly used probiotic strains, the ability of cellulose sulphate encapsulation to protect from low pH in artificial gastric juice containing pepsin, followed by treatment with bile. CS encapsulation offers exceptional protection also for strains thought previously to be acid resistant such as *L. casei shirota* and *L. acidophilis* [43, 44]. *L. casei* is afforded more than 8 logs protection by cellulose sulphate encapsulation, whilst *S. boulardii* is afforded around 5 logs protection. As compared to chitosan encapsulation, CS encapsulation gave a 10,000 fold better protection for *L. casei* and a 100,000 fold better protection than alginate plus gelatinized starch after 3 h exposure to simulated human gastric fluid [45]. The CS capsules used in this study have pores that allow larger molecules than H^+^ ions to enter and leave the capsules [46]. The internal CS material carries an excess of negatively charged sulfate groups and it is possible that these charged groups buffer the bacteria from the harmful effect of stomach acid by preventing high concentrations of H^+^ ions from entering the capsule.

In our study, viable *E. coli-LUX* (auto-fluorescing *E. coli* expressing luciferase) were used to follow the transit and release of bacteria in the gastric tract. These bacteria were chosen because they are commensal and colonize the gastric tract of mice and humans [47–49]. This particular strain had been shown in previously published studies to efficiently colonize the gastric tract of mice [26]. In the study described here, viable *E. coli-LUX* were detectable in both the cecum and colon of mice orally gavaged with encapsulated bacteria. In contrast, almost no *E. coli-LUX* were detected in mice orally gavaged with free, non-encapsulated bacteria. The difference was especially noticeable in the colon (Fig. 7e, f). Further, more than 1 log more *E. coli-LUX* were detected in mouse fecal pellets 2, 4 and 24 h after ingestion of orally gavaged encapsulated bacteria compared to orally gavaged free, non-encapsulated bacteria (Fig. 7e, f), suggesting that not only had the bacteria survived the 4–6 h transit through the gut but had been released and colonized the gastric tract as evidenced by the high levels of expression detected in the feces 24 h after gavage.

Release is most probably a result of a combination of the low levels of cellulase found in the lower gastric tract and the peristaltic movement. The digestibility of cellulose and hemicellulose was previously estimated at around 70% in a group of seven women on a standardised diet [50] showing that there is extensive degradation of these polysaccharides in dietary plant cell wall material during passage through the human intestine. However, in the same study only 8% of an added refined cellulose (Solka Floc) was digested showing that the type of cellulose is apparently critical [50]. This is supported by the finding that bacteria able to grow on sources of hydrated, amorphous cellulose, such as spinach cell walls, can apparently be isolated from most individuals whereas bacteria that degrade largely crystalline cellulose substrates, such as milled filter paper, are not always recoverable [51–53]. The bacterial strains isolated from human feces that are able to digest cellulose include *Ruminococcus *sp., *Clostridium *sp., *Eubacterium *sp. and *Bacteroides *sp. [51–54]. We were able to mimic this effect in vitro using equivalent concentration ranges of cellulase and gentle agitation overnight (Fig. 6 and Table 1). In this respect, it is important to note that the robustness of the capsules can be increased or decreased by modifying the encapsulation parameters.

## Conclusion

The ability to deliver individual or mixtures of members of the microbiome by the oral route, using cellulose sulphate capsules which protect extremely efficiently against low pH and proteolytic enzyme digestion over long periods, whilst releasing the bacteria in the lower intestine, would make many current probiotic treatments much more effective. One area that would also benefit is FMT which currently is complicated by the high heterogeneity of fecal samples since no two samples from different individual donors will ever be the same [55]. Efficient delivery of specific mixtures of bacteria in specific ratios, without appreciable loss, would very much simplify FMT, and make it more acceptable as well as more routine and less costly.

## Data Availability

The datasets during and/or analysed during the current study available from the corresponding author on reasonable request.
